# Insights from a dataset on behavioral intentions in learning information flow diagram capability for software design

**DOI:** 10.1016/j.dib.2023.109307

**Published:** 2023-06-10

**Authors:** Meennapa Rukhiran, Titiya Chomngern, Paniti Netinant

**Affiliations:** a Faculty of Social Technology, Rajamangala University of Technology Tawan-ok, Chanthaburi, Thailand; b College of Digital Innovation Technology (DIT), Rangsit University, Pathum Thani, Thailand

**Keywords:** Information design, Requirement development, Software design, Software development, TAM model, Structural education model

## Abstract

Developing complex software may be difficult for students or those with less technical expertise in software design due to the large number of diagrams and the complexity of their relationships. Unified modeling language (UML) provides conceptual software design as a system's blueprints, including programming statements, software processes, software components, deployment, design, and development, whereas database schemas use UML for an object-oriented database and entity relation model (ERD) for a relational database. An information flow diagram (IFD) is a technical tool for designing software that includes Infrastructure, data and information, and processing flow. IFD can benefit from examining a new paradigm that facilitates a more practical and rapid understanding of information designs. This data set contains the results of an investigation into the factors affecting the acceptance of IFD for software design by college students. Google forms are used to collect information from undergraduate and graduate computer science, IT, and software engineering students. The extended technology acceptance model (TAM) will focus on studying factors affecting acceptance or decision to use IFD, which includes the ability to create information flow diagrams, satisfaction with software design, and business requirement expectations. This study was carried out at four Thai universities. Research data collection for software design and development courses spanned the academic year 2021. Concerning the use of IFD in software design, 537 respondents were questioned regarding their perceptions, behavioral intentions, information flow diagram capability, software design satisfaction, and business requirement expectations. All students completed the survey. To ensure that participation was voluntary, each participant gave informed consent. Any collected information was rendered anonymous. The participants were given the information solely for research purposes. Ethical values, respect, autonomy, compassion, and confidentiality were guaranteed. The survey's primary questions correspond to the acceptance model's independent variables. Online distribution of the questionnaire yielded 537 valid responses. The dataset consists of 1) student demographics and 2) student perspectives on the factors influencing their intent to learn and apply IFD to software design. Partial Least Squares - Structural Equation Modelling was utilized to analyze the data (PLS-SEM). With the help of these data, researchers, software developers, and educators in various settings can use and analyze alternative software designs and validate models used to study and predict the acceptance of behaviors and factors.

**Specifications Table**

**Table:** 

Subject	Software Engineering
Specific subject area	Software design, software development, e-learning system, learning assessment, technology acceptance model (TAM), business requirement.
Type of data	Data in csv files Tables Figure
How the data were acquired	The dataset was compiled by collecting raw data through an online Google form questionnaire. The data is formatted as an Excel spreadsheet. Using SPSS 28.0 and SmartPLS 4.0.8, all valid samples were analyzed for construct validity and reliability, Exploratory Factor Analysis (EFA), Confirmatory Factor Analysis (CFA), and Structural Equation Model (SEM). As the primary factor analyses, the technology acceptance model (TAM) factors, including perceived ease of use and usefulness, were implemented. By extending the TAM model, the information flow diagram ability, software design satisfaction, and business requirement expectation could be studied to determine user behavior intention to accept a novel software design diagram using information flow design and development for software engineering.
Data format	Raw Cleaned and Analyzed Descriptive and statistical dataset
Description of data collection	Private and public university students in Thailand were the target demographic. After the software design class, students' intentions to learn information flow diagrams for two semesters, from January 2021 to February 2022, were gathered. By distributing online questionnaires, primary data sources were collected through Google Forms. The sample for this study consisted of 537 individuals with various characteristics who were required to evaluate a new software design, referred to as an information flow diagram for software design. All participants were over 18 years old and registered undergraduate-graduate students in computer science, information technology, and software engineering programs studying software design-related courses. The authors conducted, distributed, and collected a survey using a sample of 537 students from four private and public universities who completed questionnaires. No invalid response forms were submitted during the survey. Region: Asia, Kingdom of Thailand
Data source location	Authors’ survey, with sample of 537 students from four private and public universities Region: Asia City: Pathum Thani, Chonburi, Chanthaburi Country: Thailand
Data accessibility	Repository name: Mendeley Data Data identification number: 10.17632/nvnz4d44m2.3 Direct URL to data: https://data.mendeley.com/datasets/nvnz4d44m2/3
Related research article	*-*

## Value of the Data

• The dataset is essential for validating that a technical tool of a single software design known as an information flow diagram can better influence software design and development for a convenient, rapidly concise understanding of information designs related to Infrastructure, information, and flow processes.
• The data present the data analysis for evaluating students' behavioral intent to accept the information flow diagram's materials during the software design phase of the software development life cycle [1]. The data is beneficial in addressing the factors that influence the introduction of a novel new study by introducing innovative factors based on the study via the technology acceptance model (TAM) and structural equation model (SEM).
• The data benefits all parties involved, particularly those involved in software design and development, higher education students and teachers in computer science and information technology, and those involved in software engineering education and instruction.
• These primary data, factors, and questionnaires can be used for further research into existing/new learning and teaching methodologies in software engineering of higher education. In the future, our research will be completed with greater specificity and objectivity from the perspectives of many nations.

## 1 Objective

Software Development Life Cycle (SDLC) is a software paradigm [2] for defining and analyzing business requirements and software processes via business modeling. Building an appropriate SDLC is difficult for a specific type of information system design since a system has involved and requires careful preparation and administration. To guarantee the standard and quality of an end design delivering a robust, effective, and efficient system, the system design could support and ensure that the software engineering design is accomplished [3]. Less-experienced software developers may find it challenging to comprehend the design and development of complex software. The new paradigm of information flow diagrams (IFD) has been proposed as a technical tool for a single software design related to infrastructure, information, and flow processes. This is attained by enhancing the skills and knowledge of students. The information flow diagram represents the relational process of software design, data relations, databases, user interface (UI), interface designs, operations, processing steps, information flows, and data stores. All software elements in the design have access to and can be evolved for software development. This study investigates the student's learning skills and knowledge of software analysis and design phases based on software engineering elements, including databases, processes, users, and user interactions. This data collection is based on select constructs of the technology acceptance model (TAM) [4], such as perceived usefulness (PU), perceived ease of use (PEU), behavioral intention to use (BI), attitude (ATT), and actual use (ACT) of the new approach. Three unexplored factors of the data collection newly established to investigate higher education students' acceptance of the information flow diagram through software design in Thailand, where the significance of enhancing skills and knowledge in software engineering toward information flow diagram ability (IFDA), software design satisfaction (SWDS), and business requirement expectation (BURE).

## 2 Research Justifications

TAM is utilized in the context of IFD capability for software design because IFD is a technical tool for software development design. IFD can represent the infrastructure, data, information flow, processing flow, database, and user interfaces of software systems. Software engineering has been viewed as a process technology. The tools, techniques, and methods used to create and manage business processes represent process technology. IFD is a process technology that aids software designers and developers in visualizing information and processing flows in software systems and technologies. Consequently, user perceptions of IFD's usefulness, usability, satisfaction, and expectations can impact the tool's adoption and acceptance as a software design resource. TAM is a well-established framework for researching people's behavioral intentions and technology use, including process technologies such as IFD. This study utilized TAM's concepts and measurement scales to investigate the acceptance of IFD for software design in process technology of software design among college students. Moreover, this study contributed to the field of process technology acceptance and usage behavior by analyzing and interpreting data from the dataset, comparing and integrating findings with prior research that has used TAM in similar contexts, and thereby analyzing and interpreting data from the dataset.

## 3 Data Description

**Fig. 1:**
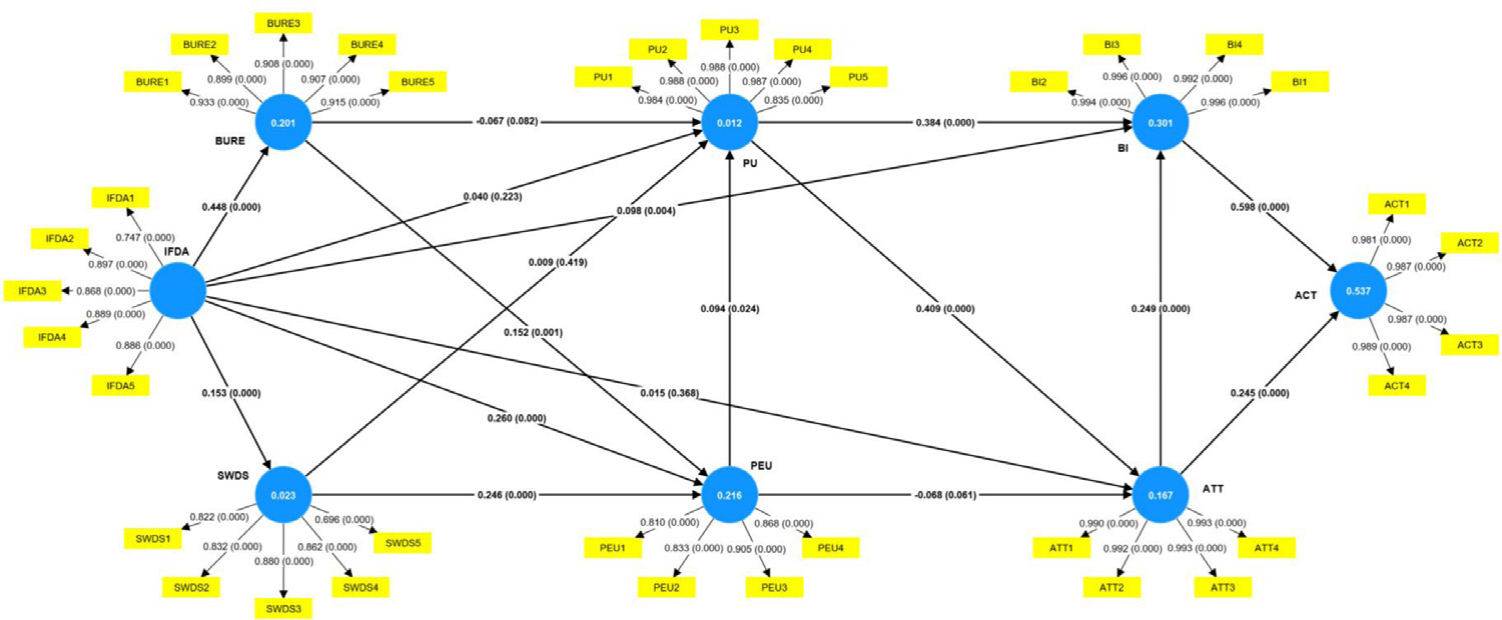
Measurement and structural model analysis.

This article's dataset contains information regarding the most recent evaluation of the information flow diagram. Table 1 displays the demographic information of students who learned the information flow diagram in university settings. The level of agreement with statements representing factors that influence students' acceptance of the learning information flow diagram is depicted in Table 2. The construct factors consist of perceived usefulness (PU), perceived ease of use (PEU), behavioral intention to use (BI), attitude (ATT), and actual use (ACT) of the new approach involved in this study, the importance of enhancing skills and knowledge in software engineering toward information flow diagram ability (IFDA), software design satisfaction (SWDS), and business requirement expectation (BURE). The response scales of PU, PEU, ACT, IFDA, and SWDS employed a seven-point Likert scale, where 'strongly agree' was scored as 7, 'agree' as 6, 'slightly agree' as 5, 'either' as 4, 'lightly disagree' as 3, 'disagree' as 2, and 'strongly disagree' as 1. The five-point Likert scale is less confusing and increases the response rate [5] due to the BI and ATT constructions. Cronbach's alpha is shown in Table 3 to represent the construct's reliability and validity. Internal consistency of the original questionnaire was determined to be acceptable (Cronbach's alpha > 0.70). As demonstrated in Table 4, the heterotrait-monotrait ratio of correlations (HTMT) criterion measures the average correlations of the indicators across constructs. Table 5 displays the constructs' discriminant validity as measured by the Fronell-Larcker criterion [6]. Table 6 illustrates Cross loading among constructs’ analysis to confirms the reliability of constructs. Table 7 provides a data analysis of the relationship between variables for each acceptance criterion factor for the information flow diagram in classes. Fig. 1 depicts the bootstrapping result of SEM in SmartPLS 4.0 for user acceptance of information flow diagrams in the software design domain [7].

**Table 1:** Respondents’ characteristics.

Demographic	Characteristics	Frequency	Proportion (%)
Gender	Male	308	57.40
	Female	229	42.60
Age	Between 18-21 years old	177	33.00
	Between 22-24 years old	271	50.50
	Between 25-29 years old	73	13.60
	Between 30-34 years old	7	1.30
	More than 34 years old	9	1.70
Major	Computer Sciences	114	21.20
	Information Technology	329	61.30
	Software Engineering	94	17.50
Education Level	Undergraduate	438	81.60
	Graduate	99	18.40
University	Rangsit University	99	18.40
	Rajamangala University of Technology Tawan-OK	102	19.00
	Bangkok University	210	39.10
	Walaialongkorn University	126	23.50

**Table 2:** Descriptive results of students’ response to the research survey (*N* = 537).

Variables	Items	Range	Min	Max	Mean	S.D.	Variance
ACT	I plan to use IFD for software design.	5	2	7	4.20	1.530	2.340
	I will continue to use IFD in software development.	5	2	7	4.21	1.573	2.474
	I would agree that IFD can be applied for my future project and career.	5	2	7	4.21	1.540	2.373
	I would recommend introducing IFD in software engineering class.	5	2	7	4.22	1.564	2.446
ATT	It is necessary to learn IFD to improve skills and knowledge in software engineering class.	3	2	5	3.75	0.805	0.648
	I support to use IFD in software design phase.	3	2	5	3.74	0.813	0.661
	I have a positive view to learn IFD in class.	3	2	5	3.75	0.818	0.670
	A development team supposes to use IFD in software design.	3	2	5	3.77	0.832	0.692
BI	I think using IFD for analysis and design is the right choice.	3	2	5	3.82	0.949	0.901
	I predict I will use IFD.	3	2	5	3.79	0.950	0.903
	I intend to use IFD.	3	2	5	3.81	0.957	0.915
	I think I will introduce IFD to others.	3	2	5	3.80	0.935	0.874
BURE	A single IFD can reach business requirement as expected.	5	2	7	6.21	1.084	1.175
	IFD design can readily be used throughout the entire analysis and design process.	5	2	7	6.07	1.026	1.053
	The performance of IFD can support analysis, design, and development in early and later stages.	5	2	7	6.15	1.059	1.121
	If a development team used IFD for software design, the team can decrease time to develop software.	6	1	7	6.43	1.031	1.063
	A software productivity will increase based on user requirements if the team use IFD.	5	2	7	6.42	1.089	1.185
IFDA	The ability of IFD represents to design attribute and concern.	3	4	7	6.73	0.640	0.409
	The ability of IFD represents to design constraint and element.	4	3	7	6.55	0.841	0.707
	The ability of IFD represents to design entity and overlay.	4	3	7	6.53	0.848	0.720
	The ability of IFD represents diagrams associated to Infrastructures, Information Flows, and User Interface)	3	4	7	6.41	0.871	0.758
	The ability of IFD represents to design stakeholder and subject.	4	3	7	6.58	0.754	0.569
PEU	Illustrating IFD in design process is easy and understanding for me.	5	2	7	4.44	1.420	2.015
	IFD is clear and understandable for software design viewpoints.	5	2	7	4.45	1.447	2.095
	I find IFD easy to learn and use for software design.	5	2	7	4.44	1.392	1.937
	It is easy for new learners to improve skills and knowledge in software design when using IFD.	5	2	7	4.44	1.432	2.049
PU	Single design of IFD can represent the whole software elements for software development phase.	3	2	7	4.08	0.829	0.688
	IFD can help me accomplish design viewpoints more quickly.	5	2	7	6.11	1.195	1.427
	IFD can increase my skills and knowledge in software design.	6	1	7	5.53	1.444	2.086
	A single design of IFD is useful to demonstrate user interface design.	4	3	7	5.92	1.060	1.124
	A single design of IFD is useful to demonstrate software functionalities.	5	2	7	5.91	1.138	1.295
SWDS	I am satisfied to learn IFD for design software.	5	2	7	6.08	1.220	1.489
	I am satisfied to learn IFD for developing software.	6	1	7	5.51	1.462	2.138
	I am satisfied to use IFD for design software.	4	3	7	5.89	1.068	1.141
	I am satisfied to use IFD for developing software.	5	2	7	5.87	1.159	1.342
	I am satisfied to recommend IFD to others.	6	1	7	5.88	1.258	1.584

**Table 3:** Construct reliability and validity.

Constructs	Cronbach's Alpha	Composite Reliability (RHO_A)	Composite Reliability (RHO_C)	Average variance extracted (AVE)
ACT	0.990	0.991	0.993	0.972
ATT	0.995	0.995	0.996	0.984
BI	0.996	0.996	0.997	0.989
BURE	0.950	0.955	0.961	0.832
IFDA	0.910	0.921	0.934	0.738
PU	0.878	0.907	0.916	0.731
PEU	0.977	0.980	0.982	0.918
SWDS	0.879	0.898	0.911	0.674

**Table 4:** Discriminant validity -HTMT.

	ACT	ATT	BI	BURE	IFDA	PU	PEU	SWDS
ACT								
ATT	0.491							
BI	0.702	0.407						
BURE	0.025	0.065	0.132					
IFDA	0.059	0.046	0.121	0.474				
PU	0.067	0.040	0.050	0.310	0.397			
PEU	0.628	0.410	0.497	0.034	0.055	0.099		
SWDS	0.046	0.021	0.018	0.136	0.167	0.339	0.038

**Table 5:** Discriminant validity - Fornell-Larcker criterion.

	ACT	ATT	BI	BURE	IFDA	PE	PEU	SWDS
ACT	**0.986**							
ATT	0.487	**0.992**						
BI	0.698	0.405	**0.994**					
BURE	0.019	0.063	0.129	**0.912**				
IFDA	0.034	0.009	0.118	0.448	**0.859**			
PU	0.032	-0.025	-0.044	0.301	0.366	**0.855**		
PEU	0.619	0.404	0.489	-0.019	0.046	0.091	**0.958**	
SWDS	0.039	-0.012	-0.013	0.131	0.153	0.306	0.036	**0.821**

**Table 6:** Cross loading among constructs’ analysis.

	ACT	ATT	BI	BURE	IFDA	PEU	PU	SWDS
ACT1	**0.981**	0.463	0.683	0.003	0.027	0.609	0.014	0.026
ACT2	**0.987**	0.495	0.681	0.036	0.045	0.610	0.048	0.035
ACT3	**0.987**	0.471	0.706	0.003	0.025	0.628	0.018	0.041
ACT4	**0.989**	0.493	0.681	0.031	0.037	0.594	0.046	0.051
ATT1	0.470	**0.990**	0.382	0.063	0.000	0.399	-0.014	-0.011
ATT2	0.483	**0.992**	0.398	0.061	0.010	0.398	-0.023	-0.015
ATT3	0.489	**0.993**	0.417	0.076	0.012	0.407	-0.032	-0.014
ATT4	0.491	**0.993**	0.409	0.051	0.015	0.399	-0.027	-0.008
BI1	0.691	0.403	**0.996**	0.130	0.124	0.486	-0.023	-0.011
BI2	0.699	0.402	**0.994**	0.127	0.111	0.493	-0.062	-0.014
BI3	0.694	0.405	**0.996**	0.125	0.121	0.484	-0.026	-0.009
BI4	0.691	0.402	**0.992**	0.131	0.113	0.483	-0.066	-0.016
BURE1	0.002	0.065	0.077	**0.933**	0.404	-0.052	0.295	0.127
BURE2	0.022	0.119	0.152	**0.899**	0.394	0.001	0.250	0.080
BURE3	0.042	0.053	0.122	**0.908**	0.407	0.024	0.309	0.140
BURE4	0.008	0.035	0.138	**0.907**	0.472	-0.039	0.284	0.130
BURE5	0.010	0.013	0.094	**0.915**	0.349	-0.020	0.221	0.110
IFDA1	0.065	0.034	0.081	0.307	**0.747**	0.017	0.239	0.136
IFDA2	0.021	0.041	0.108	0.421	**0.897**	0.051	0.325	0.125
IFDA3	-0.049	-0.075	0.047	0.345	**0.868**	-0.017	0.341	0.084
IFDA4	0.060	0.012	0.100	0.386	**0.889**	0.053	0.353	0.142
IFDA5	0.046	0.023	0.159	0.447	**0.886**	0.078	0.305	0.165
PEU1	0.624	0.393	0.492	-0.012	0.041	**0.984**	0.101	0.036
PEU2	0.617	0.399	0.475	-0.027	0.043	**0.988**	0.102	0.043
PEU3	0.615	0.391	0.474	-0.012	0.053	**0.988**	0.104	0.040
PEU4	0.623	0.393	0.476	-0.029	0.039	**0.987**	0.105	0.040
PEU5	0.475	0.358	0.424	-0.012	0.045	**0.835**	0.017	0.009
PU1	-0.048	-0.035	-0.077	0.218	0.311	0.040	**0.810**	0.213
PU2	0.038	-0.042	-0.023	0.219	0.286	0.079	**0.833**	0.235
PU3	0.016	-0.027	-0.045	0.383	0.398	0.074	**0.905**	0.295
PU4	0.110	0.025	-0.004	0.158	0.222	0.125	**0.868**	0.295
SWDS1	-0.003	-0.023	-0.032	0.128	0.154	0.036	0.295	**0.822**
SWDS2	0.064	0.017	0.007	0.027	0.085	0.008	0.230	**0.832**
SWDS3	0.020	-0.023	-0.002	0.149	0.166	0.028	0.247	**0.880**
SWDS4	0.060	-0.002	0.000	0.122	0.101	0.041	0.282	**0.862**
SWDS5	0.029	-0.015	-0.024	0.089	0.109	0.029	0.172	**0.696**

## 4 Experimental Design, Materials, and Methods

Prior to data collection, a qualitative research method was employed to validate the research constructs and revise the research items. The primary data in this article was collected as part of an investigation of students' behavioral intentions to learn information flow diagrams as a new software design paradigm during two semesters between March 2021 and February 2022. In Thailand's four universities, student information was collected. The students studied the same instructional materials regarding the utilization of information flow diagrams in the design of an e-learning system. Information flow design for representing flow diagrams of information, process, interaction, database, and user interfaces was introduced in software design and development courses. Students introduced information flow diagrams, compared them to other software paradigms, such as unified modeling language and data flow diagrams, analyzed and designed an e-learning system, and discussed their skills and knowledge of academic achievement. At the end of each course section, students were required to complete an online survey. The characteristics of 537 participants are shown in Tables 1 and 2, respectively. The data were analyzed using statistical tests using the PLS-SEM method implemented in Smart PLS 4.0.8 software.

**Table 7:** Summary of SEM without control variables results. Or Summary of path coefficient of variables based on SEM.

	Original Sample	Standard Deviation	T Statistics	P values	Result
ATT -> ACT	0.245	0.031	7.955	0.000	Supported****
ATT -> BI	0.249	0.035	7.160	0.000	Supported****
BI -> ACT	0.598	0.029	20.523	0.000	Supported****
BURE -> PEU	0.152	0.047	3.221	0.001	Supported***
BURE -> PU	-0.067	0.048	1.394	0.082	Supported*
IFDA -> ATT	0.015	0.044	0.338	0.368	Not Supported
IFDA -> BI	0.098	0.038	2.613	0.004	Supported***
IFDA -> BURE	0.448	0.048	9.259	0.000	Supported****
IFDA -> PEU	0.260	0.041	6.311	0.000	Supported****
IFDA -> PU	0.040	0.053	0.761	0.223	Not Supported
IFDA -> SWDS	0.153	0.042	3.667	0.000	Supported****
PEU -> ATT	-0.068	0.044	1.543	0.061	Supported*
PEU -> PU	0.094	0.048	1.978	0.024	Supported**
PU -> ATT	0.409	0.034	12.211	0.000	Supported****
PU -> BI	0.384	0.034	11.236	0.000	Supported****
SWDS -> PEU	0.246	0.048	5.095	0.000	Supported****
SWDS -> PU	0.009	0.046	0.205	0.419	Not supported

Note: SEM-PLS estimation results (*n* = 537, **** *p* < 0.001, *** *p* < 0.01; ** *p* < 0.05, * *p* < 0.1).

Bold indicates to highlight the strongly supported results.

### 4.1 Development process of construct items

The development of constructs provides a comprehensive understanding of the key factors influencing the acceptance of the information flow diagram in the software design process. The development stages of construct items were divided into night steps as follows:

(1) Identify the research constructs: The first step is to identify the key constructs or factors that the research is trying to measure. In this research, the primary constructs based on the Technology acceptance model [4] were perceived usefulness (PU), perceived ease of use (PEU), behavioral intention to use (BI), attitude (ATT), and actual use (ACT). The authors proposed a novel methodology for software engineering education using an information flow diagram. Therefore, there are essential constructs that can be designed and considered in this study as follows:
  1.1 Perceived usefulness (PU): This construct measures the degree to which students believe using the information flow diagram will enhance their performance and improve the quality of their work. It is measured using items that assess the usefulness of the information flow diagram in terms of productivity, accuracy, and ability to complete tasks more efficiently.
  1.2 Perceived ease of Use (PEU): This construct measures students' perceptions of the ease of using the information flow diagram in their software design process. It is measured using items that assess the simplicity of the information flow diagram, how user-friendly it is, and how easy it is to learn.
  1.3 Behavioral intention to use (BI): This construct measures the intention of students to use the information flow diagram in their software design process. It is measured using items that assess their willingness and desire to use the information flow diagram in the future.
  1.4 Attitude (ATT): This construct measures students' overall evaluation of the information flow diagram. It is measured using items that assess the students' positive or negative feelings toward the information flow diagram.
  1.5 Actual use (ACT): This construct measures students' actual usage of the information flow diagram in their software design process. It is measured using items that assess the frequency and duration of use of the information flow diagram.
  1.6 Information flow diagram ability (IFDA): This construct measures students' ability to use the information flow diagram in their software design process. It is measured using items that assess their skills, knowledge, and understanding of the information flow diagram.
  1.7 Software design satisfaction (SWDS): This construct measures the students' satisfaction with their software design process using the information flow diagram. It is measured using items that assess their overall satisfaction with the design process and the outcomes of their software design projects.
  1.8 Business requirement expectation (BURE): This construct measures the students' expectations of the information flow diagram's ability to meet business requirements in their software design process. It is measured using items that assess their expectations of the information flow diagram's ability to support software development that meets business requirements.
(2) Conduct a literature review: A thorough review of the existing literature related to the research constructs should be conducted to ensure that the constructs being measured are relevant and up-to-date.
(3) Determine the item content: Based on the literature review, determine the content for each construct item. The content should accurately reflect the concept being measured.
(4) Write the items: Write the items clearly and concisely, making sure to use language that is easily understood by the target population.
(5) Pretest the items: Pretest the items by giving them to a small group of people similar to the target population. This will help identify any issues with the items and ensure they are clear and easily understood.
(6) Refine the items: Based on the pretest results, make any necessary revisions to the items. This may involve rewording items, adding or removing items, or changing the response options.
(7) Pilot test the revised items: Conduct a pilot test with a larger sample to further assess their reliability and validity.
(8) Evaluate the results: Evaluate the pilot test results to determine the items' reliability and validity. Make any necessary revisions to improve the construct measures.
(9) Finalize the items: After all, revisions have been made, finalize the items and include them in the survey questionnaire.

### 4.2 Necessary of SWDS and BURE in the model

Software design satisfaction (SWDS) and business requirement expectation (BURE) are two important constructs introduced in this study to assess the students' acceptance of information flow diagrams in software design. These two factors have been included in the research model to comprehensively understand students' attitudes toward information flow diagrams and their expected outcomes from using this technical tool.

SWDS measures the students' satisfaction level with the information flow diagram as a tool in software design. This construct aims to understand how well the information flow diagrams meet the students' expectations and how well they are perceived as software design tools. The high level of SWDS implies that the students are satisfied with the information flow diagrams, which leads to positive attitudes towards the tool and increased usage.

On the other hand, BURE measures the students' expectations of the business requirements they expect to fulfill through the information flow diagram. This construct aims to understand the students' expectations of anticipated outcomes using the information flow diagrams. A high level of BURE indicates that the students have high expectations from the tool and expect a positive impact on their software design and development process.

Therefore, including SWDS and BURE in the research model provides a comprehensive understanding of students' attitudes toward information flow diagrams and the outcomes they expect from using the tool. These constructs play a crucial role in evaluating the effectiveness of information flow diagrams as a tool for software design and in understanding the students' adoption and usage patterns.

### 4.3 Measurement model

Table 3–6 illustrates convergent and discriminant validity, as well as composite reliability. In Table 3, the instrument's reliability, consistency, and validity are evaluated. As suggested by the fact that Cronbach's Alpha values are greater than 0.70 [8], the data set contained values between 0.878 and 0.990. A composite reliability (CR) value greater than 0.70 is recommended [9]. Thus, the data set contained values between 0.89 and 0.99. Hair et al. [9]   recommended that the average extracted variance (AVE) be greater than 0.50, so the data set landed between 0.674 and 0.990. Consequently, the data set indicates extremely reliable. All demonstrated acceptance of convergent validity achieved and recommended measure values (AVE > 0.5 and CR > 0.7). Tables 4 and 5 demonstrate the discriminant validity of the HTMT and the Fornell-Larcker criterion, respectively. Table 6 illustrates the cross-loading of constructions. The discriminant's validity was established because each construct's square root was more significant than their respective inter-construct correlation estimates [10], which were also significant.

The data set was processed to evaluate the coefficient of determination criteria proposed by Hair et al. [9] suggesting that an R^2^ value of 0.2 is considered a high-degree effect. The R^2^ values of BURE, PEU, BI, and ACT were 0.201, 0.216, 0.301, and 0.537, respectively, indicating a high degree of interpretation of IFDA to BURE. Consequently, IFDA, PU, and ATT to BI all have a high degree of interpretation. In conclusion, BI and ATT to ACT were highly interpretable. This data set is acceptable, as depicted in Fig. 1.

### 4.4 Assessment of PLS-SEM model

Fig. 1 depicts the use of partial least-squares structural equation modeling (PLS-SEM) to evaluate the proposed exploration model. Hair et al. [9] clarified how exploratory and confirmatory factor analysis methods could be utilized to examine multiple dependence relations concurrently and sequentially. This is especially useful when model constructs have both direct and indirect effects on one another. The initial step in interpreting PLS-SEM results is to examine the model-fit indicator, which demonstrates that the data perfectly fit the proposed model. The regression weights shown in Fig. 1 are the result of applying PLS-SEM to the proposed model. The implemented model demonstrates that all proposed relationships are supported. The characteristics of IFD usage as an acceptance of software design tools are demonstrated in Table 7. The *p*-value is less than 0.001, the *t*-value is greater than 1.96, the *p*-value is less than 0.1, and the *t*-value is greater than 1.31 (*df* = 30, *p* = 0.1), confirming the significance of the effect (*p* < 0.001, *p* < 0.01, *p* < 0.05, and *p* < 0.1) [9]. Since the path coefficient and *t*-value were performed (beta = 0.245; *t*-value = 7.955; *p* < 0.001) and (beta = 0.249; *t*-value = 7.160; *p* < 0.001), respectively, ATT had a statistically significant positive effect on ACT and BI. Since the path coefficient and *t*-value were performed (beta = 0.598; *t*-value = 20.523; *p* < 0.001), BI was found to be significantly and positively related to ACT. As the path coefficient, BURE was found to be significantly and positively related to PEU (beta = 0.152; *t*-value = 3.221, *p* < 0.01). BURE was found to be significantly and negatively related to PU (beta = -0.067; *t*-value = 1.394; *p* < 0.1). IFDA was significantly and positively associated with BI (beta = 0.098; *t*-value = 2.613, *p* < 0.01), BURE (beta = 0.448; *t*-value = 9.259, *p* < 0.001), PEU (beta = 0.260; *t*-value = 6.311, *p* < 0.001), and SWDS (beta = 0.153; *t*-value = 3.667, *p* < 0.001). Students discovered that the information flow diagram capabilities had a positive impact on business user requirements, that usability had an indirect effect on utility, and that software design was satisfied. Consequently, the data analysis confirmed that IFD is a legitimate software design tool. However, the data analysis of IFDA revealed that it was insignificant and directly related to PU and ATT. The data analysis of SWDS revealed that it was insignificant and directly related to PU.

### 4.5 Limitations of the study

The limitations of the data analyzed in the study must be taken into account when interpreting the results. The use of PLS-SEM to evaluate the proposed exploration model is one of the limitations. PLS-SEM is useful for examining multiple dependencies simultaneously and sequentially, but it may not provide a complete picture of the relationships between the constructs. Another limitation is the absence of a direct correlation between key variables, such as IFDA and PU or SWDS and PU. Although the results demonstrated that IFDA positively impacted several variables, including BI, PEU, and BURE, it had no direct effect on PU. Similarly, the results showed that SWDS had a positive impact on a number of factors but had no direct impact on PU. Notably, the findings are based on self-reported data collected from college students, which may not necessarily reflect the opinions and experiences of other populations. In addition, the study only considered a limited number of factors that may influence software design acceptance, omitting other factors that may be significant, such as personal motivations, prior experiences, and cultural influences.

## Ethics Statements

This research does not involve hazardous chemicals, equipment, procedures, animal or human testing, or the use of animals. Rangsit University has granted ethical approval, and the actual protocol number is RSU-GRAD 501/2558. Informed consent was obtained from all study participants. Participants were at least 18 years old and enrolled at four universities. As part of ethical research, the authors respect the voluntariness, anonymity, freedom, and confidentiality of the participants. The provided data contained no information that could be used to determine the participants' identities.

## CRediT authorship contribution statement

**Meennapa Rukhiran:** Conceptualization, Methodology, Software, Supervision, Writing – review & editing, Project administration. **Titiya Chomngern:** Data curation, Investigation, Writing – original draft, Validation, Project administration, Funding acquisition. **Paniti Netinant:** Visualization, Investigation, Software, Validation, Formal analysis, Supervision, Writing – review & editing.

## Declaration of Competing Interest

The authors declare that they have no known competing financial interests or personal relationships that could have appeared to influence the work reported in this paper.

## Data Availability

Dataset for Behavioral Intentions based on Learning Acceptance of Information Flow Diagram Capability for Software Design (Original data) (Mendeley Data).

## Appendix Supplementary materials

Image, application 1
