# Supplementary material for: Insights from a dataset on behavioral intentions in learning information flow diagram capability for software design
Source: Data Brief. 2023 Jun 10;49:109307. doi: 10.1016/j.dib.2023.109307 (PMC10279554; doi:10.1016/j.dib.2023.109307)
Supplement: Supplementary file 1 [file mmc1.pdf]

Informed Consent (Online Survey)

Study Title: Behavioral Intentions based on Learning Acceptance of Information Flow Diagram Capability for Software Design

We have discussed the evolution of each hypothesis. This study aims to investigate the acceptance of learning the information flow diagram capability for software design. As a technical tool for a single software design involving infrastructure, information, and flow processes, the new paradigm of information flow diagrams (IFD) has been proposed. This is accomplished by enhancing students' skills and knowledge. We are asking participants to take part in the study because you are currently pursuing a degree in computer science, information technology, or software engineering, which can be attained by enhancing student software design skills and knowledge. **As a quality measure, please answer yes or no to each of the following questions:**

1. Are you currently enrolled in courses pertaining to software design and development?
2. Do you currently pursue a bachelor's or master's degree in computer science, information technology, or software engineering?
3. Have you studied the new paradigm of information flow diagrams and conducted case studies?
4. I affirm that I am at least 18 years old.

If you answered **"no"** to any of the above questions, you do not meet the eligibility requirements for participation in this study. Please delete this email and thank you for your consideration.

If you answered **"yes"** to each of the questions, you are eligible to participate in this study.

(*NOTICE: By clicking the above link you are consenting to participate in this research survey. Please continue reading for more information.*)

Voluntary participation:

Your participation in this research study is completely voluntary. You are free to withdraw at any time from the research study. Participants can withdraw from online surveys at any time prior to completion by simply abandoning the survey. Participants in an online survey may also skip questions they do not wish to answer. To include your responses, please click "submit" at the end of the survey. Even if you choose not to participate in the study or withdraw, you will still receive the same benefits and opportunities as other students. Your decision will not affect your grade or your ability to graduate from university. You should direct any questions you may have regarding this research study to the listed principal investigator. In the future, if you do not understand something that is being done, you may ask him questions. This consent form describes the research study in which your participation is requested. Before agreeing to participate, please review this form carefully and ask any questions you may have about the study.

Procedures:

Rangsit University is conducting an online survey on your behalf as part of a research project. This research is being conducted as a program requirement for the research project of a practical enhancement of information flow diagram capability for software design and development courses in university majors in computer science, information technology, or software engineering. The University requires your consent for participation in this project. You must be 18 or older to participate in this survey.

- Assume you agree to participate in the research study. In that case, you will be asked to complete an online survey regarding your perceptions of perceived usefulness (PU), perceived ease of use (PEU), behavioral intention to use (BI), attitude (ATT), actual use (ACT), information flow diagram ability (IFDA), software design satisfaction (SWDS), and business requirement expectation (BURE). The survey also contains questions regarding your demographic information. During the first-class session, each student will be informed of the research topic, the proposal, and the methodology. After the end of the semester, this research will be collected online with the consent of the participants. We anticipate that the survey will take between 20 and 25 minutes to complete. Participants can withdraw from online surveys at any time prior to completion by simply abandoning the survey. You may choose not to respond to a specific question or any section of the survey by proceeding to the next question. Participants' responses will be included in the data analysis and report if they click the "submit" button at the end of the survey.
- Your responses will be anonymous, and your IP address will not be recorded to ensure complete anonymity. Using email or the internet may compromise privacy, confidentiality, or anonymity. In spite of this possibility, the risks to your physical, emotional, social, professional, and financial well-being are deemed "less than minimal" if you choose to complete the survey.
- Your participation in this study may not directly benefit you, but it will help us understand current opinions and demographic factors related to measuring the acceptance of the information flow diagram technique for usage and learning. We believe that this study will provide valuable insight into the actual state of software design and development. This knowledge will serve as a foundation for comprehending the factors influencing the acceptance of learning information flow diagram capability for software design and development. We sincerely hope that the data collected in this study will be used to guide software design education using information flow design and development in software engineering fields.

Risks of harm/Discomforts/Inconvenience: There are no known risks associated with participation in this study. By agreeing to participate in this research, you have not waived your right to legal recourse in case of harm caused by the research. The risks of participating in this study are no more than what is experienced in learning and using information flow diagrams for software design and development courses.

Privacy and Confidentiality:
Privacy and confidentiality of participants will be maintained throughout this study. Participants who choose to click "Cancel" or abandon the survey during the research process will have no data collected; their data will be destroyed. The electronic data (survey results) will be stored on a memory stick that is password-protected. No identities will be associated with any data resulting from this study, and all data will be kept anonymous. Information from the online survey will be coded to protect the anonymity and confidentiality of survey respondents and summarized in an anonymous format within the body of the final report. No specific comments will ever be attributed to a specific individual unless prior consent has been obtained. All documents will be held in strict confidence. January 2021 to February 2022 will be used to collect data for the anticipated study. The data collected in support of this research project will be retained and possibly granted for future studies while participant anonymity and privacy are maintained. All Students will have access to the final report once it has been completed.

If you have questions about this project, you may contact Assoc. Prof. Paniti Netinant, Ph.D. (+66) 923-936-655) email: paniti.n@rsu.ac.th
